# Base Excision Repair of Chemotherapeutically-Induced Alkylated DNA Damage Predominantly Causes Contractions of Expanded GAA Repeats Associated with Friedreich's Ataxia

**DOI:** 10.1371/journal.pone.0093464

**Published:** 2014-04-01

**Authors:** Yanhao Lai, Jill M. Beaver, Karla Lorente, Jonathan Melo, Shyama Ramjagsingh, Irina U. Agoulnik, Zunzhen Zhang, Yuan Liu

**Affiliations:** 1 Department of Environmental Health, Sichuan University West China School of Public Health, Chengdu, Sichuan, P. R. China; 2 Department of Chemistry and Biochemistry, Florida International University, Miami, Florida, United States of America; 3 Department of Cellular Biology and Pharmacology, Florida International University, Miami, Florida, United States of America; University of Pittsburgh, United States of America

## Abstract

Expansion of GAA·TTC repeats within the first intron of the frataxin gene is the cause of Friedreich's ataxia (FRDA), an autosomal recessive neurodegenerative disorder. However, no effective treatment for the disease has been developed as yet. In this study, we explored a possibility of shortening expanded GAA repeats associated with FRDA through chemotherapeutically-induced DNA base lesions and subsequent base excision repair (BER). We provide the first evidence that alkylated DNA damage induced by temozolomide, a chemotherapeutic DNA damaging agent can induce massive GAA repeat contractions/deletions, but only limited expansions in FRDA patient lymphoblasts. We showed that temozolomide-induced GAA repeat instability was mediated by BER. Further characterization of BER of an abasic site in the context of (GAA)_20_ repeats indicates that the lesion mainly resulted in a large deletion of 8 repeats along with small expansions. This was because temozolomide-induced single-stranded breaks initially led to DNA slippage and the formation of a small GAA repeat loop in the upstream region of the damaged strand and a small TTC loop on the template strand. This allowed limited pol β DNA synthesis and the formation of a short 5'-GAA repeat flap that was cleaved by FEN1, thereby leading to small repeat expansions. At a later stage of BER, the small template loop expanded into a large template loop that resulted in the formation of a long 5'-GAA repeat flap. Pol β then performed limited DNA synthesis to bypass the loop, and FEN1 removed the long repeat flap ultimately causing a large repeat deletion. Our study indicates that chemotherapeutically-induced alkylated DNA damage can induce large contractions/deletions of expanded GAA repeats through BER in FRDA patient cells. This further suggests the potential of developing chemotherapeutic alkylating agents to shorten expanded GAA repeats for treatment of FRDA.

## Introduction

Friedreich's ataxia (FRDA, OMIM 229300) is one of the most prevalent inherited autosomal recessive neurodegenerative disorders. The prevalence of FRDA is 1:50,000 in Caucasians, and it has been estimated that the carrier rate can reach as high as 1:120-1:60 [1]–[3]. The disease is caused by the silencing of the frataxin gene [4], which further results in a low level of a 220-amino acid mitochondrial protein, frataxin in cells. Because frataxin plays a crucial role in maintenance of iron homeostasis, heme biosynthesis and assembly of iron-sulfur clusters (ISCs) into metabolic enzymes [5], cellular deficiency of the protein can lead to an insufficiency of electrotransfer through a series of proteins and enzymes of the respiratory chain. This subsequently increases electron leakage that in turn results in energy deficiency and oxidative stress leading to death of large sensory neurons in the dorsal root ganglia (DRG) and the posterior columns of the spinal cord among others [6].

It has been found that frataxin gene expression is disrupted by expanded GAA repeats located in the first intron of the frataxin gene in FRDA patients [4]. The normal length of GAA repeats ranges between 6 and 36, whereas the repeats in FRDA patients can expand to up to 1700 repeat units with the majority of patients bearing 600 to 900 triplets [4], [7], [8]. The level of mature frataxin transcript and frataxin protein in FRDA patient cells is inversely correlated with the length of GAA repeats, so that longer GAA repeats lead to the lower levels of frataxin mRNA transcript and frataxin protein [9]. In addition, the extent of GAA repeat expansion correlates with disease severity and early age of onset [7], [10], [11].

Expanded GAA repeats in FRDA patients can form a variety of unusual secondary structures, including purine:purine:pyrimidine and pyrimidine:purine:pyrimidine triplexes [12]–[15] as well as sticky DNA [16], [17]. Moreover, the formation of RNA:DNA hybrid triplexes can occur during transcription. This can sequester RNA polymerase and transcription factors, impeding transcription of the frataxin gene [17], [18]. Expanded intronic GAA repeats can also cause abnormal heterochromatinization [19], [20] that subsequently leads to frataxin gene silencing. This is supported by the observation that the hallmarks of heterochromatin such as DNA methylation [21]–[24], histone deacetylation [25], [26] and methylation of histone H3 lysine 9 [27], [28] exist abundantly in the intronic GAA repeats-containing region of the frataxin gene. Thus, GAA repeat expansion can result in frataxin gene silencing, leading to a deficiency of frataxin by directly interfering with its gene transcription and/or facilitating the formation of heterochromatin at the region near the promoter of the frataxin gene.

Expanded GAA repeats exhibit somatic instability that can be age-dependent or age-independent. The mechanisms underlying repeat instability remain elusive. It appears that DNA replication [29], repair [30] and recombination [31] may play critical roles in causing GAA repeat instability. It has been found that during DNA replication, expanded GAA repeats resulted in replication fork stalling when GAA repeats were in the lagging strand templates [32]–[35]. This could in turn lead to the formation of hairpin/loop structures on the newly synthesized strand or template strand that further results in GAA repeat expansion and deletion [33]. Thus, the formation of secondary structures during DNA replication may be actively involved in modulating GAA repeat instability. Recent findings of persistent post-replicative junctions in human cells also point to the involvement of several post-replicative mechanisms, such as single-stranded DNA gap repair and/or double-stranded DNA break (DSB) repair-mediated recombination in modulating GAA repeat instability [36]. DSB repair in the context of GAA repeats resulted in repeat deletions through end resectioning by single-stranded exonuclease degradation of the repeats at the broken ends, or through removal of repeat flaps that were generated by homologous pairing [37]. This suggests that DSB repair is a common mechanism that resolves replication stalling caused by expanded GAA repeat tracts. This is further supported by a finding showing that GAA repeat-induced recombination was involved in chromosome fragility that is present in the human genome, including in the frataxin gene [38]. In addition, expanded GAA repeat tracts can be deleted by more than 50 bp via non-homologous end joining of DSB intermediates during DNA replication [39]. However, the age-dependent somatic instability of GAA repeats in post-mitotic non-dividing tissues, such as dorsal root ganglia [40], argues against a role for DNA replication in modulating GAA repeat instability in these tissues. Several lines of evidence have indicated that DNA mismatch repair (MMR) may mediate somatic GAA repeat expansion. It was shown that the absence of Msh2 or Msh6 proteins significantly reduced progression of GAA repeat expansion in the DRG and cerebellum in FRDA transgenic mice [41]. Ectopic expression of MSH2 and MSH3 in FRDA fibroblasts led to GAA repeat expansion in the native frataxin gene, whereas knockdown of either MSH2 or MSH3 gene expression using shRNA impeded the expansion [42]. In addition, it has been found that more MSH2, MSH3 and MSH6 proteins are expressed in FRDA pluripotent stem cells (iPSCs) that exhibit a high level of GAA instability than in their parental fibroblasts [43], [44]. Moreover, gene knockdown of either MSH2 or MSH6 in FRDA iPSCs leads to a reduced rate of GAA repeat expansions [43], [45], which is consistent with the reduced somatic GAA repeat expansions observed in the FRDA transgenic mice with their Msh2 or Msh6 gene deleted [41]. This further indicates that mismatch repair promotes somatic GAA repeat expansions.

Currently adopted strategies for FRDA treatment are aimed at correction of mitochondrial dysfunction through the use of a variety of antioxidants and iron chelators [1], [46], and intervention of heterochromatin-mediated gene silencing via histone deacetylase (HDAC) inhibitors [47]. However, the effectiveness of these therapeutic strategies is limited by expanded GAA repeats of FRDA patients although they can ease the neurodegenerative symptoms to some extent. A more effective therapy for the disease needs to be developed. Interestingly, it has been found that an expanded GAA repeat tract in peripheral blood cells and sperms of some FRDA patients may be reverted back to the normal size range by an unidentified mechanism [48], [49]. This suggests that deletion or shortening of expanded repeats can be employed as a new effective treatment for FRDA. Thus, understanding the mechanisms underlying GAA repeat contraction/deletion may help develop effective therapeutic strategies that can shorten or delete expanded large GAA repeat tracts, thereby restoring a normal level of frataxin gene expression in DRG.

Trinucleotide repeats (TNRs) including GAA repeats are tandem repeats containing guanines, which are hotspots of DNA base damage such as alkylated and oxidized base lesions. A linkage between DNA damage and somatic CAG and CTG repeat contraction/deletion and expansion has been established in bacteria [50], mammalian cells [51], [52], and mouse models [53], [54]. Furthermore, it has been found that CAG repeat expansion and deletion can be induced by the oxidized base lesion 8-oxoguanine (8-oxoG) and mediated by DNA base excision repair (BER) [52]–[57], a robust mechanism that combats the adverse effects of oxidative DNA damage. Our previous studies have demonstrated that CTG repeat instability is induced by the oxidative DNA damaging agents, bromate, chromate and H_2_O_2_ with a tendency towards contraction, and is mediated by BER of base lesions at different locations within CTG repeat tracts in human cells [52]. This suggests that BER of DNA base lesions at various locations can be actively involved in somatic deletion of any type of TNRs. Because frataxin deficiency is directly associated with elevated cellular oxidative stress in FRDA patients [58], [59], this may lead to an increased production of reactive oxygen species (ROS) that in turn generates oxidized DNA base lesions. We reason that oxidized DNA base lesions may account for the age-dependent somatic instability of GAA repeats. Moreover, because somatic deletion of expanded TNRs induced by DNA base lesions may lead to the shortening of the expanded repeats, it is possible that DNA damage-induced somatic TNR deletion can be used as a new strategy for treatment of TNR-related neurodegeneration such as FRDA. Thus, we further hypothesize that DNA base lesions induced in expanded GAA repeat tracts can result in GAA repeat deletion through BER. To test this hypothesis, we have investigated whether BER of alkylated DNA base lesions induced by the chemotherapeutic agent temozolomide in the context of GAA repeats can induce deletion of expanded GAA repeats in FRDA patient cells.

Temozolomide is an imidazoterazine-class chemotherapeutic alkylating agent that is currently used for the treatment of anaplastic astrocytoma and newly diagnosed glioblastoma [60]. It causes cancer cell death by inducing DNA base lesions, including N^7^-MeG (>70%), N^3^-MeA (9.2%) and O^6^-MeG (5%), through methylation at the N7 position of guanine, the N3 position of adenine, and the O6 position of guanine [61]. It has been found that the majority of temozolomide-induced base lesions, N^7^-MeG and N^3^-MeA, are subjected to the BER pathway [62], [63], and this has been demonstrated as one of the major mechanisms responsible for temozolomide chemotherapeutic resistance [64]. Thus, BER plays a crucial role in repairing temozolomide-induced DNA base lesions. Here, we hypothesize that temozolomide can induce multiple N^7^-MeGs and N^3^-MeAs in a long GAA repeat tract in the genome of FRDA patient cells, which can be repaired by BER. This would lead to repeat deletions, thereby shortening GAA repeats. We found that temozolomide predominantly led to large contractions, with only small expansions, in the intronic GAA repeats of FRDA lymphoblasts. We further demonstrated that the large repeat deletion was caused by the formation of a large template loop structure along with a long GAA repeat flap during BER. This allowed DNA polymerase β (pol β) to bypass the template loop and flap endonuclease 1 (FEN1) to remove the long flap resulting in a large deletion of GAA repeats. Our results reveal a mechanistic basis for large GAA repeat deletions during BER of alkylated DNA damage induced by a chemotherapeutic agent and suggest a novel strategy of using BER-dependent GAA repeat deletion for FRDA treatment.

## Materials and Methods

### Materials

Temozolomide was purchased from Sigma-Aldrich (St. Louis, MO). RPMI 1640 medium with 2.05 mM L-glutamine and fetal bovine serum (FBS) were purchased from Thermo Fisher Scientific (Pittsburgh, PA). DNA oligonucleotides were synthesized by Integrated DNA Technologies Inc. (Coralville, IA) and Eurofins MWG Operon (Huntsville, AL). The radionucleotide [γ-^32^P] ATP (6000 Ci/mmol), cordycepin 5'-triphosphate 3'- [α-^32^P] (5000 Ci/mmol) and deoxycytidine 5'-triphosphate [α-^32^P] (3000 Ci/mmol) were purchased from PerkinElmer Inc. (Boston, MA). Micro Bio-Spin 6 chromatography columns were from Bio-Rad (Hercules, CA). Deoxynucleoside 5′-triphosphates (dNTPs) were from Roche Diagnostics (Indianapolis, IN). T4 polynucleotide kinase and terminal nucleotidyltransferase were from Fermentas (Glen Burnie, MD). Mung Bean Nuclease was from Epicenter (Madison, WI). Normal-melting point agarose (NMPA), low-melting point agarose (LMPA), and ethidium bromide were from Amresco (Solon, OH). All other reagents were purchased from Sigma-Aldrich (St. Louis, MO) and Thermo Fisher Scientific (Pittsburgh, PA). Purified recombinant human apurinic/apyrimidinic endonuclease 1 (APE1), pol β, FEN1, and DNA ligase I (LIG I) were generous gifts from Dr. Samuel H. Wilson at the National Institute of Environmental Health Sciences, National Institutes of Health (Research Triangle Park, NC) or were expressed and purified as described previously [65].

### Oligonucleotide substrates

DNA oligonucleotide substrates containing a tetrahydrofuran (THF), an abasic site analog were designed to mimic an abasic site that occurs in a (GAA)_20_ repeat tract or random DNA sequence. A THF residue was used in this study because it is refractory to the 5′-deoxyribosephosphate lyase activity of pol β. Thus, its repair can only be subject to the long-patch BER sub-pathway that is involved in mediating TNR instability after single-stranded DNA (ssDNA) breaks are generated [56]. For a (GAA)_20_ repeat-containing substrate, the guanine of the tenth GAA repeat was substituted with a THF residue, whereas for a substrate containing a random DNA sequence, the twenty-third nucleotide was substituted by a THF residue. Substrates were constructed by annealing an oligonucleotide with a THF residue to its template strand at a molar ratio of 1:2. The sequences of the oligonucleotides are shown in Table S1.

### Cell culture

Human lymphoblast cell lines GM02152 (a normal individual) and GM16207 (FRDA patient with 280/830 GAA repeats) were purchased from Coriell Institute for Medical Research (Camden, NJ) and cultured in RPMI 1640 Medium supplemented with 15% fetal bovine serum and 2.05 mM L-glutamine at 37°C under 5% CO_2_.

### Measurement of instability in intronic GAA repeats in the normal and FRDA patient lymphoblasts

Instability of intronic GAA repeats induced by temozolomide was determined by treating 4×10^6^ lymphoblasts derived from a normal individual as well as a FRDA patient with expanded GAA repeats with 10 μM temozolomide for 2 hr. A concentration of 10 μM temozolomide was chosen because at this dosage the drug allowed ≥80% cell viability in the normal and FRDA patient lymphoblasts. This ensured that enough cells would be viable at the end of the experiments to isolate sufficient genomic DNA from lymphoblasts for repeat sizing analysis. Cells were then washed twice with phosphate-buffered saline (PBS), supplied with fresh medium, and grown for 2 days to allow DNA damage repair. The treatment was repeated three times before cells were harvested. At the end of the experiments, cells were harvested by centrifugation at 3000 rpm for 10 min. Genomic DNA were isolated from cells using Wizard genomic DNA purification kit (Promega, Madison, WI), dissolved in TE buffer (10 mM Tris-HCl, pH 7.5, and 1 mM EDTA), and stored at −20°C for subsequent size analysis. Untreated cells served as a negative control. The experiments were repeated at least 3 times.

### Measurement of ssDNA breaks induced by temozolomide using alkaline single cell gel electrophoresis (comet assay)

The alkaline comet assay was conducted according to the procedure described previously with minor modifications [66]. Briefly, cells were seeded in 6-well plates at a density of 10^6^ per well and treated with increasing concentrations of temozolomide for 2 hr. Cells were then collected by centrifugation at 1500 rpm for 3 min. Subsequently, 20 μl of cell suspensions were added into 80 μl 0.7% low-melting point agarose pre-warmed at 37 °C to make cell-agarose mixture, which was then spread onto a fully frosted microscope slide pre-coated with 0.8% normal-melting point agarose. After the agarose solidified, the slides were immersed in freshly prepared lysis buffer (2.5 M NaCl, 100 mM EDTA, 10 mM Tris, pH = 10.0, 1% sodium sarcosinate, 1% Triton X-100, 10% DMSO) in the dark at 4 °C for 1 hr. The slides were then soaked in the electrophoresis buffer (300 mM NaOH, 1 mM EDTA, pH>13.0) for 30 min in the dark to allow DNA unwinding. Subsequently, the slides were subjected to electrophoresis for 30 min at 0.75 V/cm. Slides were then washed with distilled water, stained with 40 μl of ethidium bromide (20 μg/ml), and analyzed under a fluorescence microscope (Carl Zeiss, Oberkochen, Germany) at 200× magnification. All the procedures were performed under the dimmed light to prevent additional DNA damage. For each treatment, 200 cells were randomly selected and scored to calculate the comet rate (%) according to the equation: comet rate (%)  =  total number of comet cells/200 counted cells, whereas 30 comet cells were randomly chosen for analyzing the Olive Tail Moment (OTM) [67] with Comet Assay Software Project (CASP, Free Software Foundation, Boston, MA). In our study, necrotic and apoptotic cells were excluded according to the criteria described by Olive and Banath [68]. The average and standard error (S.E.) or standard deviation (S.D.) of comet rate and OTM were obtained from three independent experiments.

### Measurement of BER capacity in the normal and FRDA lymphoblasts

The normal lymphoblasts and FRDA lymphoblasts with expanded GAA repeats were grown to near confluence. Cells were harvested by centrifugation at 3000 rpm for 10 min and washed twice with PBS. Cell extracts were made as described previously [69] and were dialyzed into BER reaction buffer containing 50 mM Tris-HCl, pH 7.5, 50 mM KCl, 0.1 mM EDTA, 0.1 mg/ml bovine serum albumin, and 0.01% Nonidet P-40. Substrates (25 nM) that contained a THF residue in the random DNA sequence were pre-incubated with 50 nM purified APE1 at 37°C for 30 min, and completely converted into ssDNA break intermediates for subsequent BER reactions. *In vitro* BER of a THF residue using FRDA lymphoblast cell extracts was performed by incubating APE1 precut substrates with 60 μg cell extracts at 37°C for 30 min in a 25-μl reaction mixture that contained BER reaction buffer with 5 mM Mg^2+^, 50 μM dNTPs and 50 μM [α-^32^P] dCTP. The reactions were terminated by transferring to 95°C for 10 min in 25 μl of stopping buffer containing 95% formamide and 2 mM EDTA. Subsequently, the total 50 μl of reaction mixture were applied to Micro Bio-Spin 6 chromatography columns and centrifuged at 3200 rpm for 5 min to remove the unincorporated [α-^32^P] dCTP. Repair products were then separated by 15% urea-denaturing polyacrylamide gel electrophoresis [70] and detected by a Pharos FX Plus PhosphorImager from Bio-Rad. The full length (115 nt) of random DNA sequence without any base lesion was ^32^P-labeled and run in parallel in a DNA sequencing gel to indicate the size of the repair products.

### *In vitro* reconstituted BER assay

BER of an abasic lesion (a THF residue) in the context of (GAA)_20_ repeats was performed by incubating 50 nM purified APE1, 10 nM pol β, 10 nM FEN1, and 5 nM LIG I with 25 nM (GAA)_20_ repeat-containing substrate with a THF residue. The 20-μl reaction was reconstituted with the indicated concentrations of BER enzymes and the substrate in BER reaction buffer that contained 50 μM dNTPs, 5 mM Mg^2+^ and 2 mM ATP. Reaction mixtures were assembled on ice, and incubated at 37°C for 15 min. Reactions were then terminated by transferring to 95°C for 10 min. To isolate repair products, the template strand of the substrate was biotinylated at the 5′-end. Repair products were incubated with avidin agarose beads (Pierce-Thermo Scientific, Rockford, IL) in binding buffer that contained 0.1 M phosphate, 0.15 M NaCl, pH 7.2 and 1% Nonidet P-40 at 4°C for 2 h with rotation. The agarose beads were centrifuged at 5000 rpm for 1 min and were washed three times with the binding buffer. The repaired strands were then separated from their template strands through incubation in 0.15 M NaOH for 15 min with rotation under room temperature and centrifugation at 5000 rpm for 2 min. Repaired strands were then precipitated with ethanol, dissolved in TE buffer, and stored at -20°C for subsequent size analysis.

### Probing of secondary structures by Mung Bean Nuclease digestion

Formation of secondary structures on the template and damaged strands of (GAA)_20_-containing substrates was probed by Mung Bean Nuclease digestion. Substrates (200 nM) containing a THF residue within (GAA)_20_ repeat tracts were pre-incubated with 10 nM APE1 at 37°C for 30 min to generate the ssDNA break intermediates, followed by digestion with 2 U Mung Bean Nuclease at 37°C for 1, 3, 5, 10 and 15 min. The 10-μl reaction was conducted in reaction buffer containing 30 mM sodium acetate (pH 4.6), 50 mM NaCl, 1 mM zinc acetate, and 0.01% Triton X-100. Enzymatic reactions were terminated by 2 μg proteinase K digestion at 55°C for 30 min. Reaction mixtures were subjected to 95°C for 10 min to denature the DNA. Substrates and digestion products were separated by 15% urea-denaturing PAGE and detected by a PhosphorImager. Synthesized DNA size markers were used to indicate the size of nuclease cleavage products.

### Enzymatic activity assay

Pol β DNA synthesis during BER was examined by using 25 nM oligonucleotide substrates containing (GAA)_20_ with a THF residue as illustrated in Table S1. Pol β DNA synthesis in the absence or presence of FEN1 was examined at 37°C for 15 min in a 20-μl reaction mixture that contained BER reaction buffer with 50 μM dNTPs and 5 mM Mg^2+^. FEN1 cleavage activity in the presence of pol β was examined under the same conditions used for determining pol β activity. Pol β DNA synthesis and FEN1 cleavage during BER of a base lesion in the context of (GAA)_20_ repeats were also determined in 1 min, 3 min, 5 min, 10 min, and 15 min time intervals with the (GAA)_20_ repeat substrate ^32^P-labeled at the 5′- or 3'-end. Reaction mixtures were then subjected to 95°C for 10 min in 20 μl of stopping buffer containing 95% formamide and 2 mM EDTA to denature DNA. Repair intermediates and products were separated by 15% urea-denaturing PAGE and detected by a PhosphorImager. Synthesized DNA size markers were run in parallel to indicate the size of pol β synthesized products and FEN1 cleavage products.

### Sizing analysis of GAA repeats by DNA fragment analysis and GeneMapper software

Genomic DNA isolated from temozolomide treated human lymphoblasts were amplified by a forward primer (5′- GCC AAC ATG GTG AAA CCC AGT ATC-3′) and a reverse primer tagged by a 6-carboxyfluorescein (5′-6-FAM- CCA CGC CCG GCT AAC TTT TC-3′) using the Long Range PCR kit from New England Biolabs (Ipswich, MA). Amplification of normal and expanded GAA repeats were obtained by using the following PCR procedure: 94°C for 20 s, 65 °C for 2 min (annealing and extension), 20 cycles; 94°C for 20 s, 65 °C for 2 min in which the length of this step was increased by 15 s per cycle (annealing), 65 °C for 1.5 min (extension), 17 cycles; and final extension at 65 °C for 30 min. The length of PCR products should be (79+3n) bp (n =  number of GAA triplets). Repair products resulting from *in vitro* BER in the context of (GAA)_20_ repeats were amplified by PCR with a forward primer (5′-CGA GTC ATC TAG CAT CCG TA-3′) and a reverse primer tagged by a 6-carboxyfluorescein (5′-6-FAM-CA ATG AGT AAG TCT ACG TA-3′). PCR amplification was performed under the following conditions: 95°C for 10 min, 1 cycle; 95°C for 30 s, 50°C for 30 s and 72°C for 1.5 min, 35 cycles; 72°C for 1 hr. The 6-carboxyfluorescein-labeled PCR products were then subjected to capillary electrophoresis. The size of repair products was determined by DNA fragment analysis (Florida International University DNA Sequencing Core Facility) with GeneMapper version 4.0 software (Applied Biosystems, Foster City, CA, USA). Size standards, MapMarker 1000 and 400–2000 (Bioventures, Murfreesboro, TN) were run in parallel with PCR-amplified repair products.

### Statistical Analysis

Statistical analysis was performed using GraphPad Prism 6 (Graphpad software, San Diego, CA). Significant differences in the data were examined by standard two-way analysis of variance with Tukey's multiple comparison posttests. The significant difference was designated at *P* < 0.05.

## Results

### Temozolomide induced large contractions and limited expansions in the intronic GAA repeats of FRDA patient lymphoblasts

**Figure 1:**
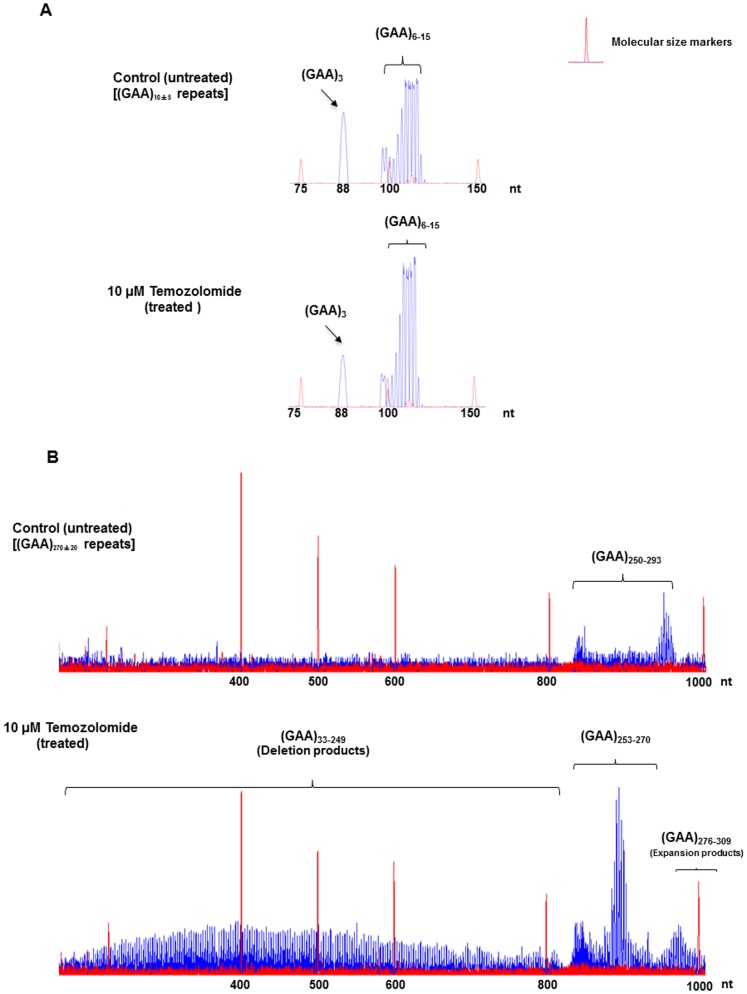
GAA repeat instability induced by temozolomide in the normal and FRDA lymphoblasts. (A) Lymphoblasts derived from a normal individual were treated with 10 μM temozolomide according to the procedure described in the Materials and Methods. Panel **A** represents the result from untreated cells. Panel **B** represents the result from the cells treated with 10 μM temozolomide. (**B**) FRDA patient lymphoblasts that contained 280 GAA repeats were treated with 10 μM temozolomide according to the procedure described in the Materials and Methods. Panel **A** illustrates the result from untreated cells. Panel **B** represents the result from temozolomide treated cells. DNA fragments including repaired products are illustrated as individual peaks. The height of a peak represents the abundance of a repair product with a specific size. The sizes of repair products are illustrated in nucleotides. Size standards are indicated.

To determine whether alkylated DNA base lesions induced in the intronic expanded GAA repeat tracts can result in GAA repeat instability, we initially examined the effects of temozolomide on the instability of intronic GAA repeats in lymphoblasts from both a normal individual and a FRDA patient. We found that temozolomide failed to induce any length change in the intronic GAA repeats of the non-patient cells (Figure 1A, panel B). The GAA repeats exhibited the same length as those in the untreated lymphoblasts that varied between 3–15 repeat units (Figure 1A, panel A). For FRDA patient lymphoblasts that contained 280 GAA repeats (GM16207), the length of GAA repeats varied from 250 to 293 repeat units (Figure 1B, panel A). Surprisingly, exposure of FRDA patient cells (GM16207) to 10 μM temozolomide led to production of a series of GAA repeat-containing fragments with 33–249, 253–270 and 276–309 repeat units, which correspond to large deletion, unaltered and small expansion products, respectively (Figure 1B, panel B). The results indicate that temozolomide predominantly induced large repeat deletions, but only induced limited expansions (up to 16 repeat units) in patient lymphoblasts (Figures 1B, panel B). Thus, we conclude that temozolomide mainly induced GAA repeat contractions in long intronic GAA repeats in FRDA patient lymphoblasts.

### Temozolomide induced ssDNA breaks in lymphoblasts from a normal individual and FRDA patient

**Figure 2:**
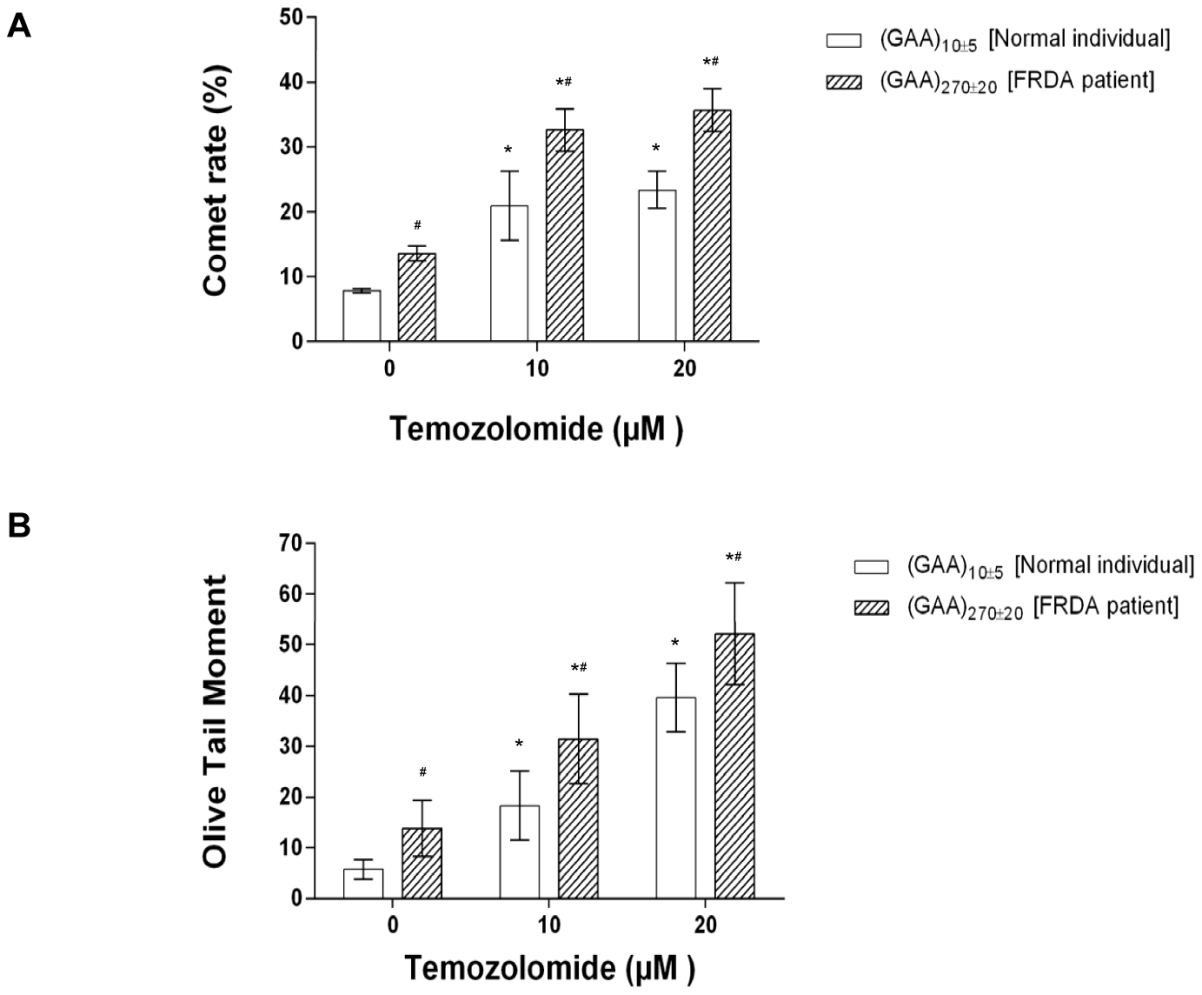
Accumulation of ssDNA breaks induced by temozolomide in the normal and FRDA lymphoblasts. ssDNA breaks in the normal individual lymphoblasts bearing intronic 15 GAA repeats and FRDA patient lymphoblasts bearing intronic 280 GAA repeats untreated or treated by 10 μM and 20 μM temozolomide, were measured using a comet assay under an alkaline condition according to the procedure described in the Materials and Methods. Comet rate (**A**) and Oliver Tail Moment (OTM) (**B**) were calculated and measured. Comet rate was calculated by the equation: Comet rate (%)  =  (total number of comet cells/total number of counted cells) ×100. All comet rates were obtained from three independent experiments and expressed as mean ± S.E. The OTM values were expressed as mean ± S.D. from 30 individual comet cells. Two-way ANOVA with Tukey's multiple comparison posttests was used to determine statistically significant differences. "*" denotes *P* < 0.05, compared to untreated cells, and "#" denotes *P* <0.05, compared with normal lymphoblasts at the corresponding concentration of temozolomide.

Because more than 80% of temozolomide-induced base lesions are N-methylated bases that can be recognized and removed by *N*-methylpurine DNA glycosylase (MPG) [64], temozolomide-induced base lesions are mainly subjected to BER, during which removal of an alkylated DNA base produces an abasic site that is subsequently cleaved by APE1 leaving a 1nt-gapped DNA (i.e. an ssDNA break intermediate). Pol β DNA synthesis then fills the single nucleotide gap, generating a nick for ligation by LIG I or a complex of DNA ligase IIIα and X-ray repair cross-complementing protein 1 (XRCC1) [63]. We reason that ssDNA breaks induced by temozolomide can lead to DNA slippage, which further results in GAA repeat instability through BER. We initially asked whether temozolomide can result in accumulation of ssDNA break intermediates in FRDA lymphoblasts. To address this question, we determined the level of temozolomide-induced ssDNA breaks in the normal and FRDA lymphoblasts by the comet assay under alkaline conditions (pH >13). This assay is a fast, simple and sensitive approach for detecting ssDNA breaks and alkali labile sites, such as an abasic site, at the single cell level. We found that exposure of both normal and FRDA patient lymphoblasts to 10 μM or 20 μM temozolomide for 2 hr significantly increased the comet rate and OTM value (*P*<0.05) (Figure 2), indicative of accumulation of ssDNA break intermediates in the cells. In addition, the comet rate and OTM value were significantly increased with increasing concentrations of temozolomide, indicating that the accumulation of ssDNA breaks was temozolomide-dependent (Figure 2). Interestingly, both untreated and temozolomide-treated FRDA patient lymphoblasts exhibited significantly higher comet rate and OTM value than non-patient cells (*P*<0.05) (Figure 2) indicating that more ssDNA breaks and alkali labile sites accumulated in FRDA lymphoblasts than in non-patient cells. This is consistent with previous findings that FRDA patient cells exhibited elevated oxidative stress due to the deficiency of the mitochondrial protein, frataxin [70], [71].

### BER of the normal and FRDA patient lymphoblasts efficiently repaired a DNA base lesion

**Figure 3:**
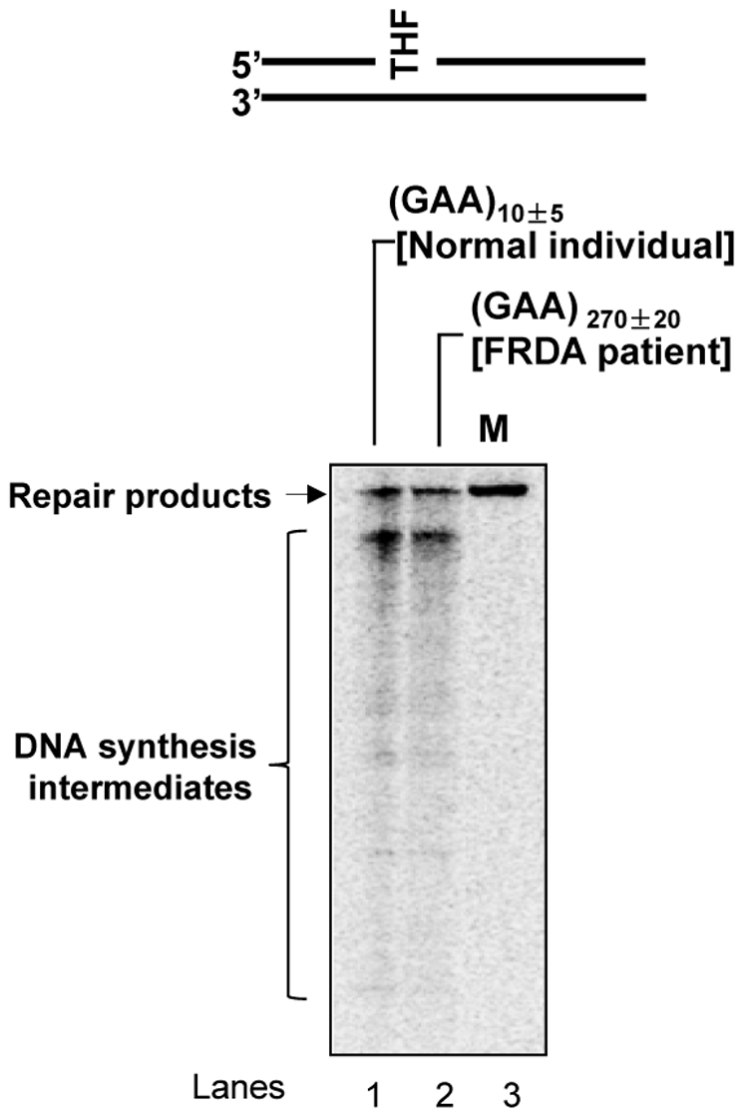
BER capacity of the normal and FRDA patient lymphoblasts. BER capacity of the normal and FRDA patient lymphoblasts was measured by incubating a random DNA sequence substrate containing an abasic site (a THF residue) with cell extracts of the normal and FRDA patient lymphoblasts under the conditions described in the Materials and Methods. Lane **1** corresponds to reaction mixture with cell extracts of the normal lymphoblasts. Lane **2** corresponds to reaction mixture with cell extracts from FRDA patient lymphoblasts with indicated GAA repeat length. Lane **3** corresponds to synthesized size marker (M).

Because temozolomide significantly increased the level of ssDNA breaks in both the normal and FRDA lymphoblasts, we then asked if the DNA damage could be efficiently repaired by BER in these cells. To address this, we measured BER capacity in both the normal and FRDA patient lymphoblasts *in vitro* by incubating cell extracts of lymphoblasts with a synthesized oligonucleotide substrate with an abasic site analog, the THF residue. The results revealed that cell extracts from both the normal and patient lymphoblasts efficiently converted ssDNA break intermediates into a repaired product (115 nt) (Figure 3). We failed to observe a significant difference in the amount of repair products between the normal (Figure 3, lane 1) and patient cells (Figure 3, lane 2) indicating that the FRDA patient cells exhibited the same BER capacity as the normal cells. Collectively, our results indicate that ssDNA break intermediates induced by temozolomide can be efficiently repaired by BER in FRDA lymphoblasts.

### BER of an abasic lesion in the context of GAA repeats leads to large deletions along with small expansions

**Figure 4:**
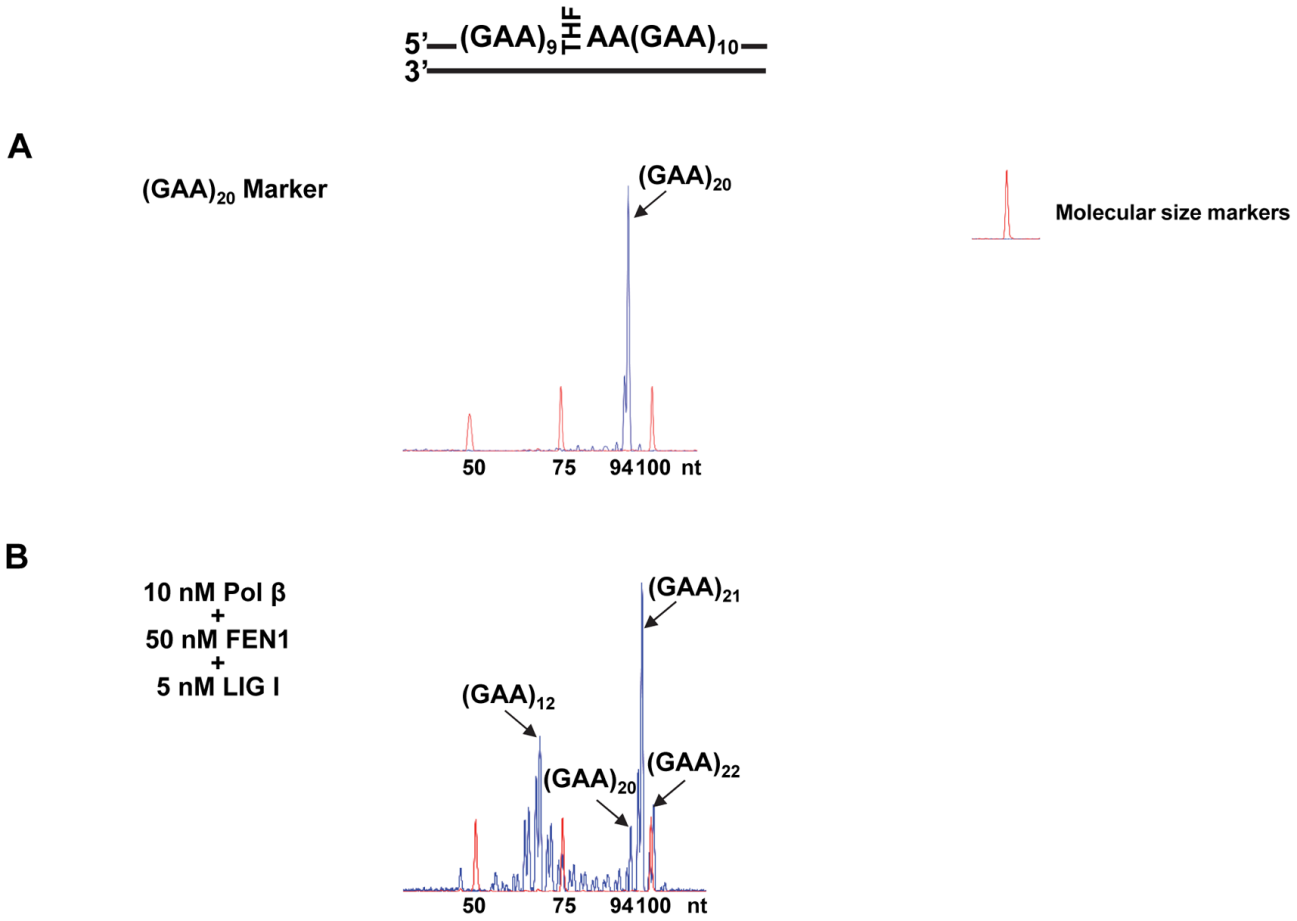
BER of an abasic lesion in the context of (GAA)_20_ repeats led to large GAA repeat deletions and small repeat expansions. The repair of an abasic lesion (THF residue) in (GAA)_20_ repeats was performed by reconstituting BER with purified BER enzymes as described in the Materials and Methods. Repaired products were then isolated and amplified by PCR and were subjected to capillary electrophoresis and DNA fragment analysis. Panel **A** illustrates the results of DNA fragment analysis of a (GAA)_20_ repeat-containing marker without any DNA damage. Panel **B** represents the DNA fragment analysis results of repair products from the reconstituted BER. Size standards are indicated.

To determine whether BER of an abasic lesion and its-resulting ssDNA break intermediates can further result in GAA repeat deletion and expansion, we examined the effects of an abasic site on the instability of (GAA)_20_ repeats during BER. A (GAA)_20_ repeat substrate with an abasic lesion (THF residue) that substituted for the guanine of the tenth GAA repeat was employed to mimic the scenarios wherein a ssDNA break occurs in the middle of a (GAA)_20_ repeat tract. BER of the abasic lesion was reconstituted with purified APE1, pol β, FEN1 and LIG I. The results revealed that the repair of a base lesion mainly resulted in a deletion of 8 GAA repeats along with an expansion of 1–2 repeats (Figure 4, panel B). PCR amplification of a (GAA)_20_ repeat-containing marker without any damage gave no repeat expansion or deletion products (Figure 4, panel A) indicating that the deletion and expansion products were DNA base lesion- and repair-dependent. The results further demonstrate that the expansion and deletion products were from BER rather than from PCR artifacts. Thus, our results indicate that BER of a base lesion in the context of GAA repeats results in large GAA repeat deletion and limited GAA repeat expansion.

### Formation of single-stranded loops on the damaged and template strands of a (GAA)_20_ repeat tract during BER

**Figure 5:**
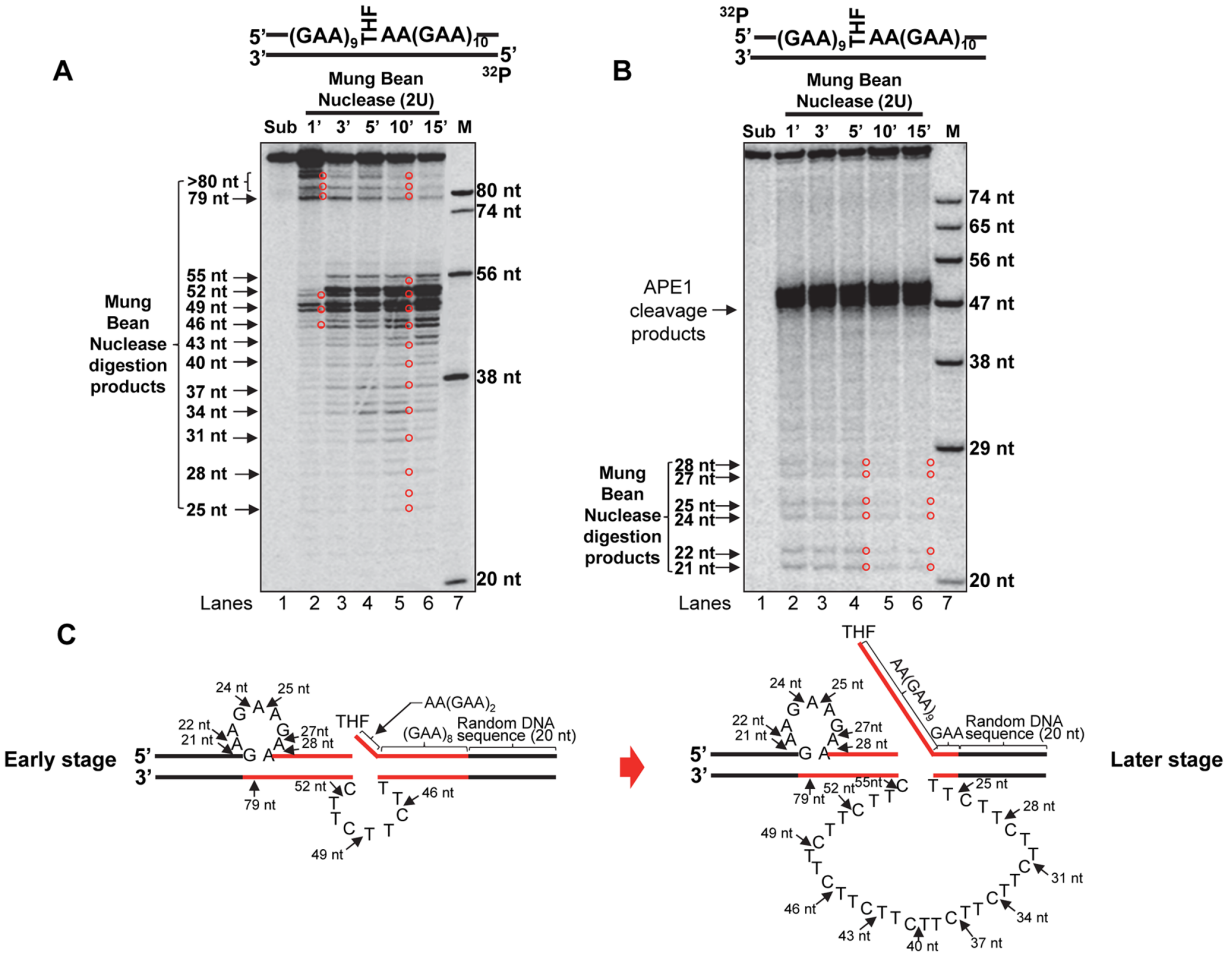
Formation of single-stranded loop structures on the damaged and template strands during BER of an abasic lesion in a (GAA)_20_ repeat tract. The secondary structures formed on the damaged and template strands of the (GAA)_20_ repeat-containing substrate with an abasic lesion located in the middle of the repeat tract, were probed by Mung Bean Nuclease digestion under the conditions described in the Materials and Methods. (**A**) Formation of secondary structures on the template strand was probed by incubating 2 U Mung Bean Nuclease with the substrate radiolabeled at the 5'-end of the template strand at various time intervals. Lane **1** represents the undigested substrate. Lanes **2−6** represent the digestion products generated at various time intervals. Lane **7** represents synthesized size markers (M). (**B**) Formation of secondary structures on the damaged strand was probed by incubating Mung Bean Nuclease with the substrate radiolabeled at the 5'-end of the damaged strand at various time intervals. Lane **1** represents the substrate only. Lanes **2−6** represent the Mung Bean Nuclease cleavage products generated at various time intervals. Lane **7** represents synthesized size markers (M). (**C**) Deduced loop structures formed on the damaged and template strands of the (GAA)_20_ repeat substrate during BER are illustrated schematically below the gels. Mung Bean Nuclease digestion sites are indicated.

Because trinucleotide repeats instability is caused by the formation of secondary structures such as hairpins and tetraplexes [72], [73], and our previous studies indicate that the formation of hairpin structures on the template and damaged strands of a (CTG)_20_ repeat tract leads to the instability of CTG repeats during BER [52]. We therefore asked if there is a secondary structure that can form in the context of GAA repeats to predominantly result in GAA repeat deletion during BER given that G and A cannot base pair with each other via a Watson-Crick base pairing. To address this we used Mung Bean Nuclease, an enzyme that preferentially makes a cleavage at a single-stranded DNA region, to determine the formation of secondary structures on the damaged and template strands of the (GAA)_20_ repeat substrate after APE1 incision of a THF residue in the GAA repeat tract. We found that Mung Bean Nuclease cleavage on the template strand resulted in products with 25 nt, 28 nt, 31 nt, 34 nt, 37 nt, 40 nt, 43 nt, 46 nt, 49 nt, 52 nt and 55 nt (Figure 5A, lane 5). Interestingly, we found that at an early time interval of 1 min, the nuclease cleavage mainly resulted in a product with 79 nt and two products that were larger than 80 nt as well as a 49 nt product (Figure 5A, lane 2). At later time intervals of 3–15 min, the nuclease cleavage generated products with 52 nt and 55 nt as well as products with 49 nt, 46 nt, 43 nt, 40 nt, 37 nt, 34 nt, 31 nt, 28 nt and 25 nt (Figure 5A, lanes 3–6). The cleavage pattern indicates that a small GAA repeat loop formed upstream of the abasic lesion of the damage strand and a small TTC repeat loop formed on the template strand at early stage of BER. During the later stage of BER, a large (TTC)_11_ loop formed on the template strand (Figure 5C). Thus, it appears that the formation of the small loop on the upstream damaged strand initiated the formation of the small and large template loops. To further confirm this, we probed secondary structures on the upstream damaged strand. The results revealed that during the first 1 min interval, Mung Bean Nuclease cleavage mainly resulted in products with 21 nt, 22 nt, 24 nt, 25 nt, 27 nt and 28 nt (Figure 5B, lanes 2–6). This confirmed the formation of a small (GAA)_3_ loop upstream of the abasic lesion in the (GAA)_20_ repeat tract on the damaged strand during the early stage of BER (Figure 5B, lane 2, and Figure 5C). The results also indicate that the formation of the small GAA repeat loop is sustained through the entirety of BER, because the nuclease cleavage products continue to exist until the later time of repair of 10 min and 15 min (Figure 5A and 5B, lanes 5–6).

### Pol β DNA synthesis and FEN1 flap cleavage during BER of an abasic lesion in a (GAA)_20_ repeat tract

**Figure 6:**
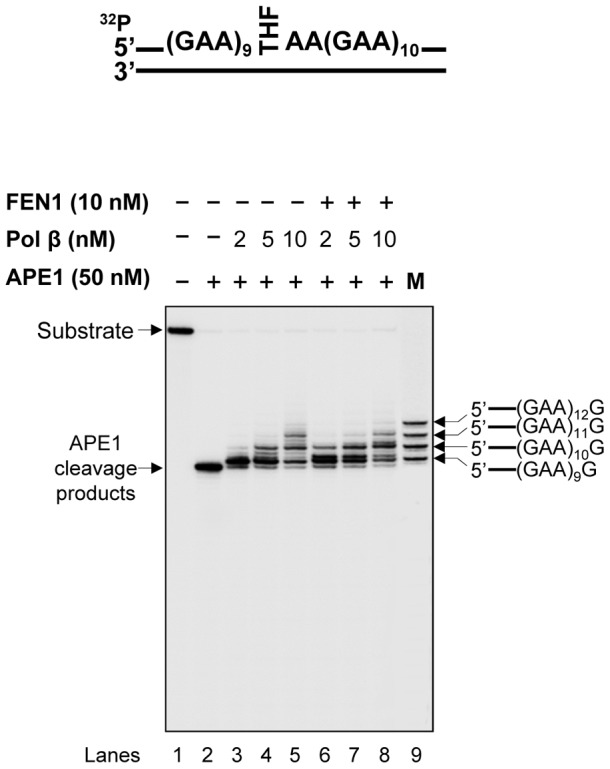
Pol β DNA synthesis during BER of an abasic lesion in the context of (GAA)_20_ repeats. Pol β DNA synthesis with the (GAA)_20_ substrate containing an abasic lesion (a THF residue) was determined by incubating the (GAA)_20_ substrate radiolabled at the 5'-end of the damaged strand with increasing concentrations of pol β in the absence or presence of 10 nM FEN1. Lane **1** corresponds to the substrate only. Lane **2** corresponds to reaction mixtures with 50 nM APE1. Lanes **3**–**5** correspond to reaction mixtures with 2, 5, and 10 nM pol β in the absence of FEN1. Lanes **6**–**8** correspond to reaction mixtures with 2, 5, and 10 nM pol β in the presence of 10 nM FEN1. Lane **9** corresponds to synthesized size markers (M). The substrate is illustrated schematically above the gel.

**Figure 7:**
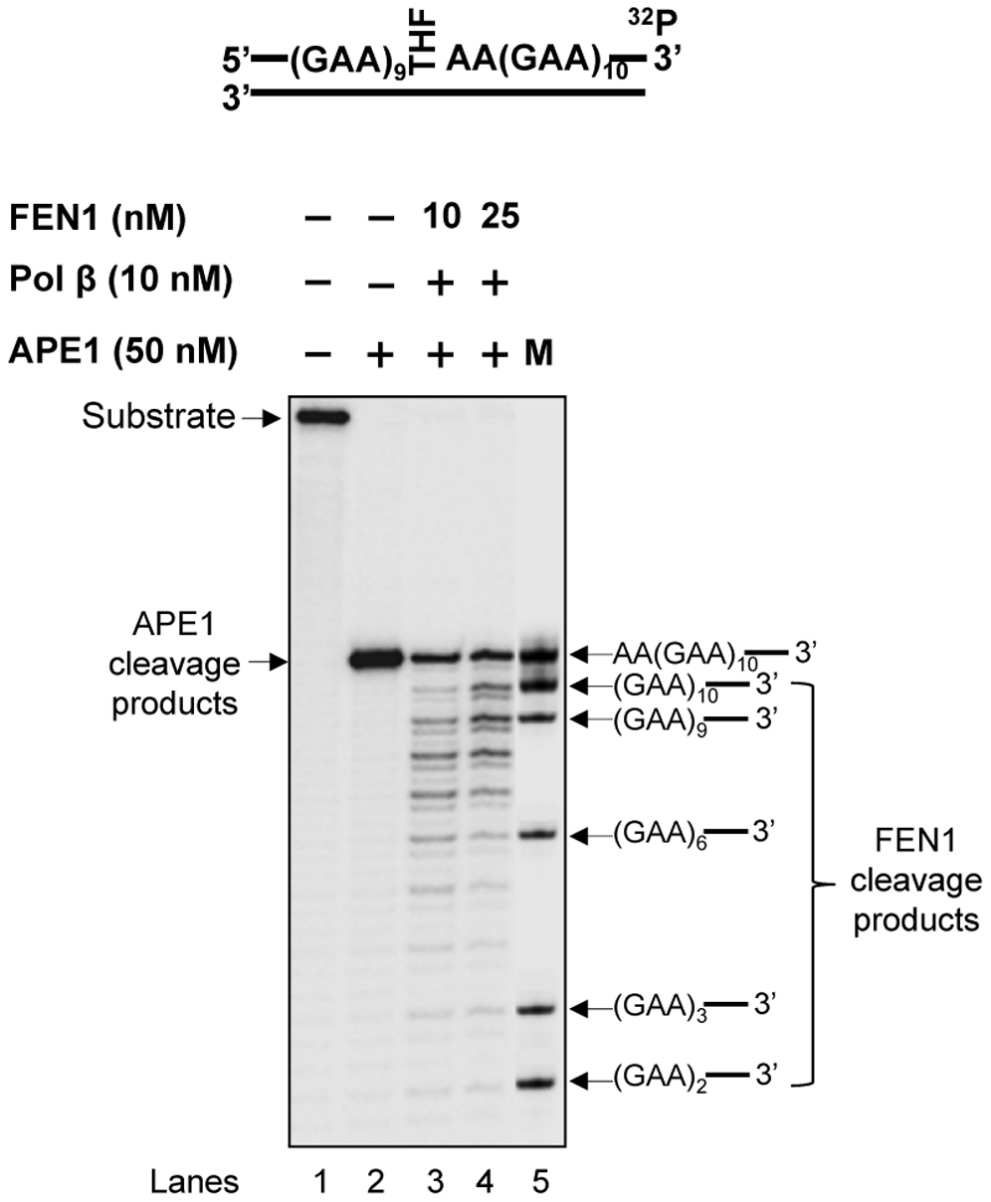
FEN1 cleavage during BER of an abasic lesion in the context of (GAA)_20_ repeats. FEN1 cleavage activity during repair of an abasic lesion in the context of GAA repeats was determined by incubating the (GAA)_20_-THF substrate with 10 nM and 25 nM FEN1 under the conditions described in the Materials and Methods. Lane **1** corresponds to the substrate only. Lane **2** corresponds to reaction mixtures with 50 nM APE1. Lanes **3−4** correspond to reaction mixtures with 10 nM and 25 nM FEN1 in the presence of 10 nM pol β. Lane **5** corresponds to synthesized size markers (M). The substrate was ^32^P-labeled at the 3'-end of the damaged strand and illustrated schematically above the gel.

**Figure 8:**
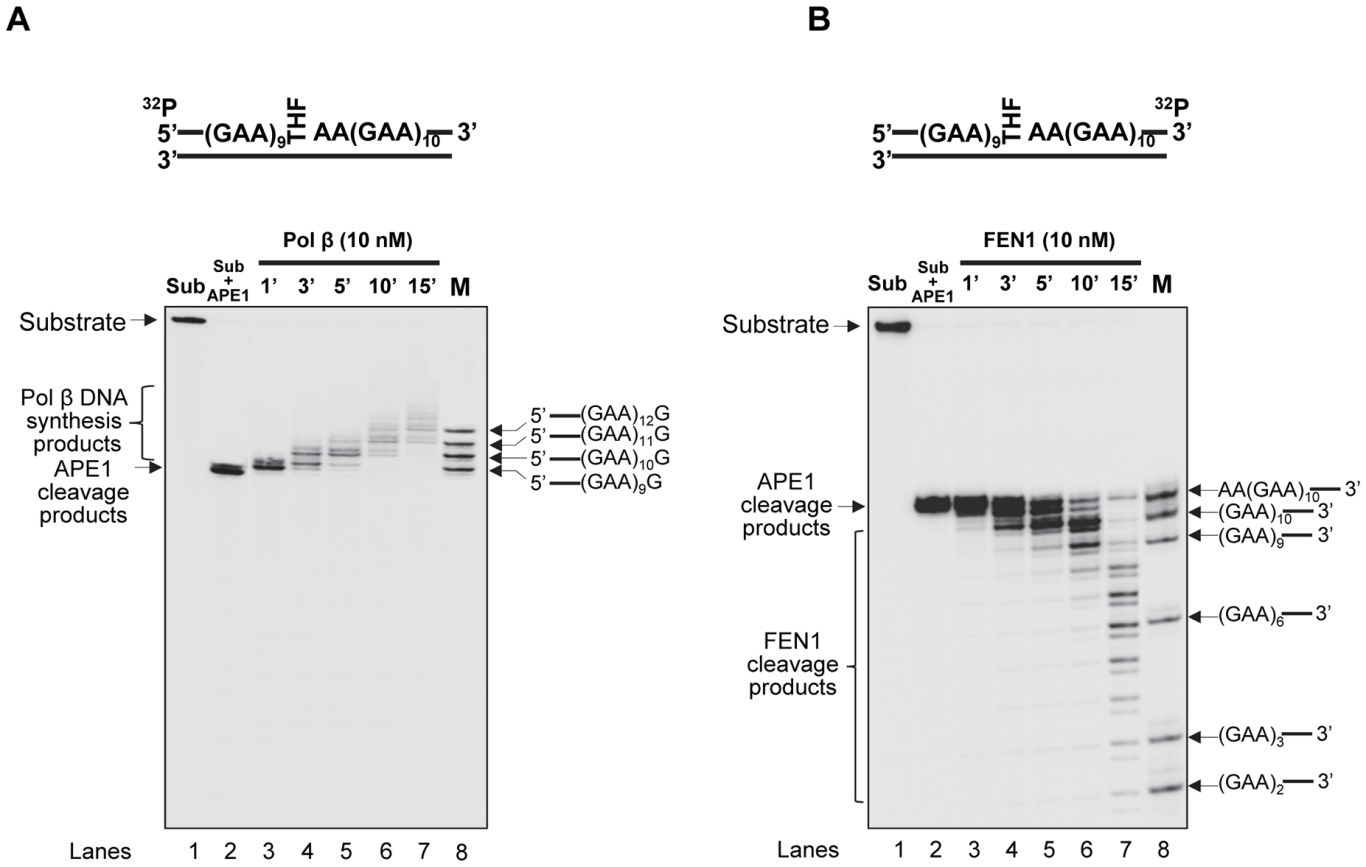
Pol β DNA synthesis and FEN1 cleavage at various time intervals during BER of an abasic lesion in a (GAA)_20_ repeat tract. (**A**) Pol β DNA synthesis during repair of an abasic lesion in the context of (GAA)_20_ repeats at different time intervals were conducted as described in the Materials and Methods. The substrate was ^32^P-labeled at the 5'-end of the damaged strand. Lane **1** corresponds to the substrate only. Lane **2** corresponds to the substrate that was pre-cut by 50 nM APE1. Lanes **3−7** correspond to reaction mixtures with APE1 pre-cut substrate, 10 nM pol β and 10 nM FEN1 at indicated time intervals. Lane **8** corresponds to synthesized size markers (M). (**B**) FEN1 cleavage during repair of an abasic lesion in the (GAA)_20_-THF substrate at different time intervals were conducted as described in the Materials and Methods. The substrate was ^32^P-labeled at the 3'-end of the damaged strand. Lane **1** corresponds to the substrate only. Lane **2** corresponds to the substrate that was pre-cut by 50 nM APE1. Lanes **3−7** correspond to reaction mixtures with APE1 pre-cut substrate, 10 nM pol β and 10 nM FEN1 at indicated time intervals. Lane **8** corresponds to synthesized size markers (M). The substrate is illustrated schematically above the gel.

Our previous study indicates that the formation of various numbers and sizes of hairpins at different locations of a (CTG)_20_ repeat tract can result in varying efficiencies of pol β DNA synthesis and FEN1 flap cleavage, that in turn governs CTG repeat deletion or expansion [52]. It is possible that the formation of small and large GAA repeat loops on the damaged and template strands can cause small repeat expansions and large repeat deletions by modulating the efficiency of pol β and FEN1. To test this, we characterized the activities of pol β DNA synthesis and FEN1 flap cleavage during BER of an abasic site (THF residue) in the context of a (GAA)_20_ repeat tract. The results revealed that pol β mainly inserted one to three repeat units during repair of the damage in the absence and presence of 10 nM FEN1 (Figure 6, lanes 3–5 and lanes 6–8). This indicates that pol β performed limited DNA synthesis during the repair of the base lesion located in the middle of the (GAA)_20_ repeat tract. In contrast, FEN1 removed up to nine repeats during repair of the abasic lesion (Figure 7, lanes 3–4), indicating that FEN1 cleaved relatively larger lengths of repeats during BER in the context of GAA repeats. Further characterization of pol β DNA synthesis and FEN1 cleavage at different time intervals indicates that pol β synthesized 1–2 repeats during 1–5 min (Figure 8A, lanes 3–5), whereas FEN1 only removed one repeat during the same time intervals (Figure 8B, lanes 3–5). At later time intervals of 10–15 min, pol β synthesized 3–4 repeats (Figure 8A, lanes 6–7), while FEN1 removed up to 9 repeats (Figure 8B, lanes 6–7). This indicates that pol β performed limited DNA synthesis during both the early and later stages of BER. FEN1 cleaved a short GAA repeat flap at the early stage, but removed a long repeat flap at the later stage of repair. We conclude that during BER in the context of GAA repeats, pol β performed an inefficient DNA synthesis by inserting a limited number of GAA repeat units (Figure 6, lanes 3–8, Figure 8A, lanes 3–7), whereas FEN1 removed a short flap at beginning of the repair (Figure 8B, lanes 3–5), and then efficiently cleaved a relatively longer flap cleavage at the later stage of BER (Figure 7, lanes 3–4 and Figure 8B, lanes 6–7).

## Discussion

**Figure 9:**
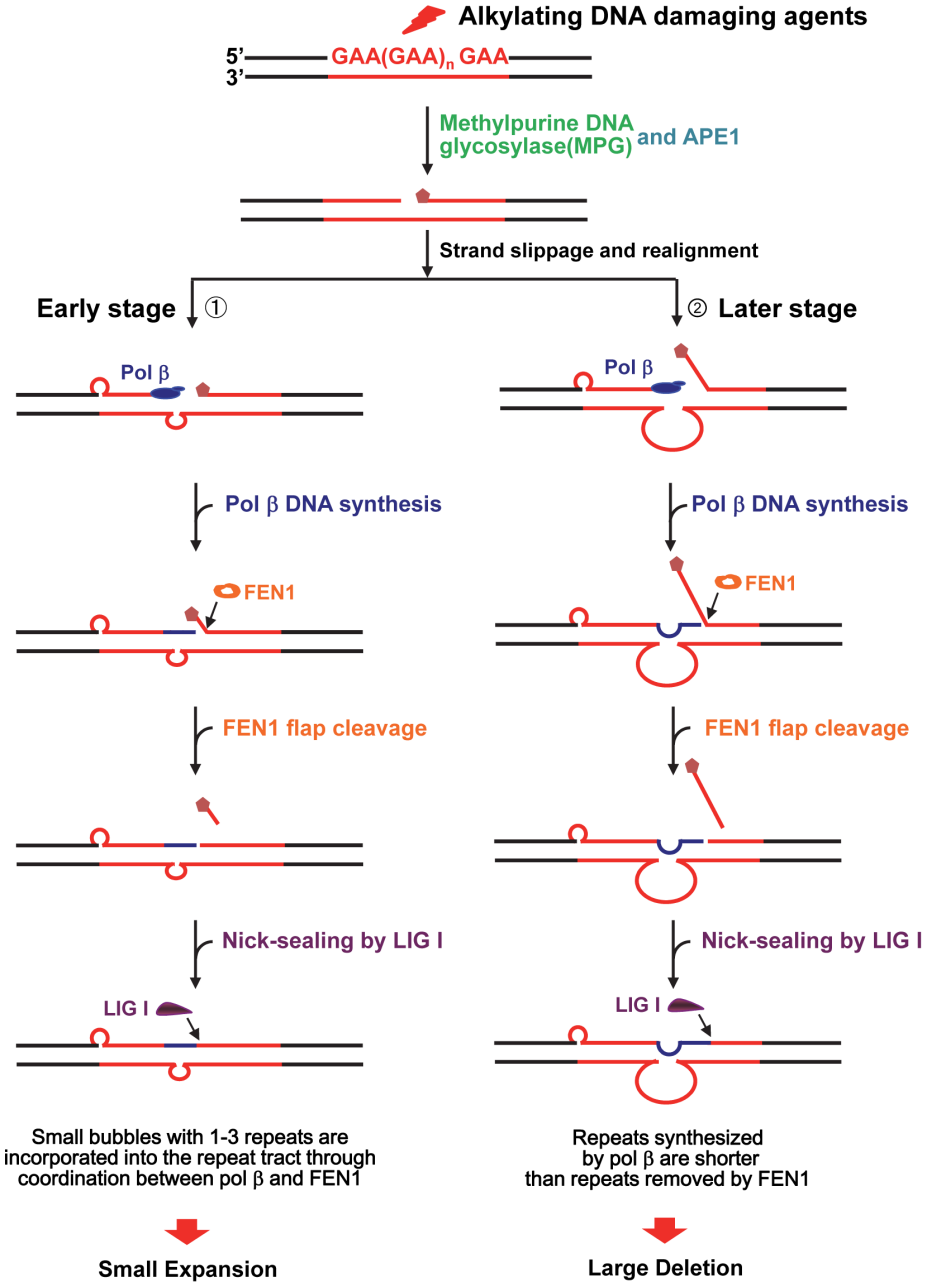
A model for large GAA repeat contractions/deletions and small expansions induced by alkylating DNA base lesions via BER. Alkylating DNA damaging agents such as temozolomide can result in methylation of a guanine and/or an adenine. Methylated bases can be removed by *N*-methylpurine DNA glycosylase (MPG). Subsequently, this leaves an abasic site that is 5'-incised by APE1 generating a ssDNA break in a GAA repeat tract. This initially causes dissociation of the upstream damaged strand and DNA slippage allowing the formation of a small upstream GAA repeat loop (Sub-pathway 1). This further results in the formation of a small template loop and a short GAA repeat flap at the downstream of the strand break. Pol β performs limited DNA synthesis, and FEN1 cleaves a short GAA repeat flap, thereby causing small repeat expansion (Sub-pathway 1). At a later stage of BER, the small template loop expands into a large loop allowing the dissociation of the downstream strand that subsequently leads to the formation of a long downstream GAA repeat flap. Pol β synthesizes a limited number of repeats to bypass the template loop. FEN1 then cleaves the long flap, resulting in a large GAA repeat contraction/deletion (Sub-pathway 2).

In this study, we provide the first evidence that the chemotherapeutic DNA damaging agent temozolomide can predominantly induce massive contractions in expanded intronic GAA repeats in FRDA lymphoblasts (Figure 1B). We demonstrate that temozolomide induces ssDNA breaks in FRDA lymphoblasts that can be efficiently repaired through BER (Figures 2–3). Further characterization on BER of an abasic lesion in the context of (GAA)_20_ repeats revealed that the repair of the base lesion resulted in a large deletion of 8 GAA repeats along with limited size (1 to 2 repeat units) of repeat expansions (Figure 4). This indicates that GAA repeat deletion and expansion was mediated by BER of base lesions in a GAA repeat tract. We further demonstrated that the large GAA repeat deletion was mediated by the formation of a large single-stranded (TTC)_11_ loop on the template strand of the (GAA)_20_ tract (Figure 5). This led to inefficient pol β synthesis of 1–4 GAA repeats (Figures 6 and 8A) and efficient FEN1 cleavage of a long (GAA)_9_ repeat flap (Figures 7 and 8B), thereby leading to a large (eight repeats) GAA repeat deletion. We showed that the small repeat expansions were mediated by the formation of a small upstream GAA repeat loop and a downstream short GAA repeat flap on the damaged strand. This led to limited pol β DNA synthesis and removal of a short repeat flap by FEN1 resulting in small repeat expansions. The results allow us to propose a model that illustrates the role of BER in mediating chemotherapeutically induced GAA repeat contractions/deletions and expansions in which an alkylated base lesion in a GAA repeat tract is removed by a damage specific DNA glycosylase, i.e., methylpurine DNA glycosylase (MPG) (Figure 9). This results in an abasic site that is 5′-incised by APE1, leaving a ssDNA break that leads to slippage of the GAA repeats and the formation of a small loop at the upstream of the ssDNA break. This subsequently triggers the formation of a small TTC repeat loop on the template strand. Pol β bypasses the small template loop by synthesizing 1 to 2 GAA repeats and creates a short downstream GAA repeat flap that is cleaved by FEN1. This leads to small GAA repeat expansions during the early stage of BER (Figure 9, Sub-pathway 1). At the later stage of BER, the small template TTC loop expands into a large loop. This further results in the formation of a long GAA flap. Pol β bypasses the template loop by synthesizing 3 to 4 GAA repeat units. FEN1 cleaves the long repeat flap removing more GAA repeats than pol β synthesizes and resulting in GAA repeat deletion (Figure 9, Sub-pathway 2).

It has been a challenge to develop an effective treatment for inherited TNR expansion-related neurodegenerative diseases. Current treatment for FRDA focuses on improvement of frataxin gene expression via altering epigenetic features at the frataxin gene [47] and the easing of the neurodegenerative symptoms [46]. However, the effectiveness of the treatment is still limited by expanded GAA repeats in the genome of FRDA patients. A strategy of shortening expanded GAA repeats should provide more effective treatment for FRDA and other TNR expansion-related neurodegenerative diseases. Thus, any strategies that can shorten expanded GAA repeats in the frataxin gene could effectively improve frataxin gene expression, thereby reducing the severity of FRDA symptoms. A study has shown that the chemotherapeutic agents mitomycin C, mitoxantrone and doxorubicin, as well as a monofunctional alkylating agent, ethylmethanesulfonate (EMS), induced deletions of expanded CTG/CAG repeats in the 5′-untranslated region (UTR) of the myotonic dystrophy protein kinase (*DMPK*) gene in myotonic dystrophy type 1 (DM1) patient lymphoblasts [51]. This suggests a potential for employing DNA damage induced TNR deletion as a target for treatment of TNR-expansion related neurodegeneration. Herein, we characterized the effects of a chemotherapeutic alkylating agent, temozolomide, on the instability of GAA repeats to explore the possibility of employing the chemotherapeutic drug as a potential treatment for FRDA. We found that temozolomide induced large contractions/deletions of expanded intronic GAA repeats in FRDA lymphoblasts, but not in a short GAA repeat tract in non-patient cells. We further demonstrated that the GAA repeat contractions/deletions were mediated by BER because temozolomide-induced alkylated DNA base lesions are mainly subjected to BER. Our results suggest that the chemotherapeutic alkylating agent, temozolomide can be developed as a potent therapeutic drug to treat FRDA via inducing alkylated base lesions and BER. It should also be noted that the GAA repeats are composed of stretches of guanines and adenines, both of which can be readily methylated by temozolomide. This could make expanded GAA repeats in FRDA patients a specific target for temozolomide-induced DNA damage treatment and enhance the effectiveness of the treatment. Moreover, as an anti-brain tumor drug, temozolomide can readily penetrate the blood-brain barrier and enter the cerebrospinal fluid [74]. It is conceivable that temozolomide can efficiently diffuse into the nerve cells in the dorsal root ganglia of FRDA patients to induce the contractions of expanded GAA repeats at a relatively low dosage. We found that 10 μM temozolomide allowed ≥80% cell survival, and can effectively contract expanded GAA repeats in FRDA patient lymphoblasts. This dose is 20-30-fold lower than the doses used for treatment of brain tumors in clinic (equivalent to ∼270–360 μM) [74]. Thus, it appears that the treatment of FRDA with the low dose of temozolomide can significantly help to reduce its side-effects, such as nausea, vomiting, headache, fatigue and anorexia [75]. Our results demonstrate a promising therapeutic effect of temozolomide on FRDA by contracting the expanded GAA repeats in the genome of FRDA patients. Our results also provide the first evidence that the temozolomide-induced GAA repeat contraction is dependent on cellular BER capacity (Figure 3) indicating a critical role for BER in a potential DNA base lesion-based treatment of FRDA.

Interestingly, we observed that Mung Bean Nuclease cleavage on the template strand of the (GAA)_20_ repeat substrate at the 1 min interval mainly resulted in large products with 79 nt and >80 nt and a product with 49 nt (Figure 5A, lane 2). This indicated that a small upstream GAA repeat loop formed on the damaged strand prior to the formation of a large loop on the template strand. This was further confirmed by the cleavage of Mung Bean Nuclease on the damaged strand that generated products 21 nt and 22 nt, 24 nt and 25 nt, as well as 27 nt and 28 nt at the first minute of BER (Figure 5B, lane 2), which indicates the formation of an upstream (GAA)_3_ repeat loop. Mung Bean Nuclease cleavage at later time intervals mainly generated products with 55 nt, 52 nt, 49 nt, 46 nt, 43 nt, 40 nt, 37 nt, 34 nt, 31 nt, 28 nt and 25 nt (Figure 5A, lanes 3–6), which indicates the formation of a large TTC loop on the template strand. Our results demonstrated a sequential order in the formation of GAA repeat loops on the damaged and template strands during BER, i.e., initially a small upstream GAA repeat loop formed at the damaged strand. This in turn triggered the formation of a small loop on the template strand that subsequently expands into a large loop (Figure 5C).

Our results also indicate that the formation of small loops on the damaged and template strands during the early stage of BER allowed pol β to synthesize 1 or 2 GAA repeats (Figure 8A, lanes 3–5). This then generated a one-GAA repeat flap that was cleaved by FEN1 (Figure 8B, lanes 3–5), thereby leading to limited repeat expansion (one or two repeat units). However, during the later stage of BER, a large TTC loop formed. This then created a large flap with 9 GAA repeats. FEN1 efficiently removed the longer flap (Figure 7, lanes 3–4, Figure 8B, lanes 6–7), whereas pol β only synthesized 3–4 GAA repeats (Figure 6, lanes 3–8, Figure 8A, lanes 6–7). This resulted in a large repeat deletion of up to 8 repeat units. These results are consistent with those showing that only limited GAA repeat expansions, but large deletions, were observed in both FRDA lymphoblasts that were treated with temozolomide (Figures 1B) and *in vitro* BER of an abasic lesion in the (GAA)_20_-containing substrate (Figure 4). Thus, our results suggest that small GAA repeat expansions occur before large GAA repeat deletions can occur during BER of base lesions induced by temozolomide. This further demonstrates a sequential production of expansion and deletion products during BER.

It has been reported that mismatch repair (MMR) proteins, MSH2, MSH3 and MSH6 are actively involved in GAA repeat expansion by binding to TNR hairpins, bulges and loops [41]–[45]. In a mismatch repair-based GAA repeat expansion model, it is proposed that during DNA replication and transcription, DNA misalignment will result in small loop-outs containing one or a few triplets that can be bound and stabilized by MutSβ (MSH2/MSH3) and/or MutSα (MSH2/MSH6). This subsequently leads to incorporation of the loop-outs into the genome causing GAA repeat expansion [42]. Multiple rounds of misalignment and MMR eventually result in the accumulation of multiple GAA repeat loop-outs that lead to large GAA repeat expansions [42]. In this study, we have discovered that BER can also be involved in somatic expansion of GAA repeats. We observed the formation of a (GAA)_3_ loop at the upstream of an abasic lesion in a (GAA)_20_ repeat tract (Figure 5) that led to a 1–2 GAA repeat expansion. It is conceivable that small GAA repeat loops formed during BER may be bound and stabilized by mismatch repair proteins leading to accumulation of multiple small GAA repeat expansions that lead to relatively large repeat expansion. This is supported by a previous finding showing that enriched binding of MSH2 and MSH3 to the intronic GAA repeats in an iPSCs derivative of FRDA fibroblasts [45], and this is associated with promotion of GAA repeat expansions in FRDA patient cells [76]. It is of importance to study the coordination between MMR and BER proteins in modulating GAA repeat instability during BER.

In this study, we have successfully developed a long-range PCR-based DNA fragment analysis method for determining the instability of TNR tracts that are longer than 135 repeats (Figure 1B). Current DNA fragment analysis can only detect trinucleotide repeat units up to 135 repeats [53]. This is because of the low efficiency of amplifying long TNR tracts by a conventional *Taq* DNA polymerase-mediated PCR. This limitation is caused by nucleotide misincorporation by *Taq* DNA polymerase, which can lead to stalling of strand extension and dissociation of the polymerase from a long repeat-containing template strand [77]. For the long-range PCR-based DNA fragment analysis method developed in our study, a DNA polymerase with 3′-5′ exonuclease activity and a *Taq* DNA polymerase were simultaneously used to carry out PCR reactions. The proofreading DNA polymerase removes the misincorporated bases, and this further allows the *Taq* polymerase to continue to synthesize DNA during amplification of long trinucleotide repeats [77]. Thus, the long-range PCR-based DNA fragment analysis provides a powerful tool to amplify and determine the size of long trinucleotide repeat tracts. Currently, the instability of TNR tracts that are longer than 135 repeats has to be determined by small-pool PCR in combination with Southern blot [40], [43], [78]. However, this approach can only roughly estimate the length of long trinucleotide repeats. Our newly developed DNA fragment analysis for long TNR tracts can provide the precise number and length changes of the repeats. In addition, our approach can detect all the possible repeat expansions and deletions of long TNRs induced by DNA damage and repair as well as other DNA metabolic pathways. Moreover, the procedure of the PCR-DNA fragment analysis is relatively simpler and faster than small-pool PCR in detecting TNR instability.

Formation of alternative secondary structures by trinucleotide repeats underlies their instability [79]. Long GAA repeats can form triplex structures and sticky DNA during DNA replication. These structures are associated with the instability of the repeats and inhibition of frataxin gene expression. However, the roles of such secondary structures in mediating GAA repeat instability remain to be elucidated. In this study, we provide the first evidence that the formation of a small upstream GAA repeat loop on the damaged strand and a large TTC repeat loop on the template strand plays an essential role in alkylated base lesions induced GAA repeat deletion and expansion. We have demonstrated that the loop structures disrupt the coordination between pol β DNA synthesis and FEN1 cleavage of GAA repeat flaps. This ultimately leads to large GAA repeat deletions and small expansions. This is also consistent with our previous studies showing that clustered hairpin structures generated in the context of CTG/CAG repeats during BER disrupt the coordination between the repair enzymes and promote inefficient BER, thereby leading to repeat deletions and expansions [52]. This further suggests that the imbalanced BER due to the formation of alternative secondary structures can be a common mechanism underlying TNR instability induced by various base lesions.

In summary, in this study, for the first time, we have demonstrated that chemotherapeutically induced alkylated DNA damage by temozolomide can predominantly lead to GAA repeat contractions in expanded intronic GAA repeats in FRDA lymphoblasts via a BER pathway. Our results indicate that during BER of a base lesion in a (GAA)_20_ repeat tract, a small upstream GAA repeat loop and a large TTC loop can form on the damaged and template strands, respectively. This further results in imbalanced pol β DNA synthesis of GAA repeats and FEN1 cleavage of the repeats, thereby causing large GAA repeat deletions, but only small repeat expansions. Our study defines a mechanism underlying alkylated DNA base lesion-induced GAA repeat contractions which is mediated by BER. We suggest that multiple rounds of DNA base alkylation lead to multiple rounds of formation of a template loop and BER that ultimately leads to large deletions of expanded intronic GAA repeats in FRDA patients. In addition, we suggest that the chemotherapeutic alkylating agent temozolomide can be potentially developed as a drug for FRDA treatment. Moreover, we have successfully developed a PCR-DNA fragment based approach to measure the instability of GAA repeats that are longer than 135 repeats.

## Supporting Information

Table S1 **Oligonucleotides sequences.**
(DOCX)
