# Supplementary material for: Base Excision Repair of Chemotherapeutically-Induced Alkylated DNA Damage Predominantly Causes Contractions of Expanded GAA Repeats Associated with Friedreich's Ataxia
Source: PLoS One. 2014 Apr 1;9(4):e93464. doi: 10.1371/journal.pone.0093464 (PMC3972099; doi:10.1371/journal.pone.0093464)
Supplement: Table S1 — Oligonucleotides sequences. (DOCX) [file pone.0093464.s001.docx]

**Table S1 Oligonucleotide sequences**

| Oligonucleotides | nt | | Sequence (5’-3’) |
| --- | --- | --- | --- |
| Substrates | | | |
| Damaged Strands | | | |
| D1 | **114** | | CGA GTC ATC TAG CAT CCG TA TC**F** CAC TGT TAT CAT GCT ATG CCT TAG GTT TTC GTG TAC TTC ATG TGT ATG TGT CAT ATA TTC ATT TGC GCT AAC TA CGT AGA CTT ACT CAT TGC |
| D2 | **99** | | CGA GTC ATC TAG CAT CCG TA GAA GAA GAA GAA GAA GAA GAA GAA GAA **F**AA GAA GAA GAA GAA GAA GAA GAA GAA GAA GAA TA CGT AGA CTT ACT CAT TGC |
| Template strands | | | |
| T1 | **115** | GCA ATG AGT AAG TCT ACG TA GTT AGC GCA AAT GAA TAT ATG ACA CAT ACA CAT GAA GTA CAC GAA AAC CTA AGG CAT AGC ATG ATA ACA GTG GGA TA CGG ATG CTA GAT GAC TCG | |
| T2 | **100** | GCA ATG AGT AAG TCT ACG TA TTC TTC TTC TTC TTC TTC TTC TTC TTC TTC TTC TTC TTC TTC TTC TTC TTC TTC TTC TTC TA CGG ATG CTA GAT GAC TCG | |
| ^a^ The damaged base is in boldface. F, tetrahydrofuran. | | | |
